# Immune Thrombocytopenic Purpura and Intracerebral Hemorrhage, Incidence, and Mortality

**DOI:** 10.7759/cureus.24447

**Published:** 2022-04-24

**Authors:** David R Hallan, Christopher Simion, Bao Y Sciscent, John Lee, Elias Rizk

**Affiliations:** 1 Neurosurgery, Penn State Health Milton S. Hershey Medical Center, Hershey, USA

**Keywords:** itp, ich, hematology, immune thrombocytopenic purpura, survival, outcomes, mortality rate, spontaneous intracerebral hemorrhage, neurosurgery

## Abstract

Introduction: Intracerebral hemorrhage (ICH) is a devastating complication of immune thrombocytopenic purpura (ITP). Using a large database, we sought to determine its incidence and mortality.

Methods: We used a de-identified database (TriNetX) to gather information on patients with ITP with subsequent ICH (cohort 1), propensity score-matched with patients with ITP and no ICH (cohort 2). Primary endpoint was mortality, with secondary endpoints of percutaneous endoscopic gastrostomy (PEG) placement, craniotomy, palliative care encounters, intensive care unit (ICU) management, seizure, falls, pulmonary embolism (PE), myocardial infarction (MI), deep venous thrombosis (DVT), ischemic stroke (IS), and other venous embolism and thrombosis (VTE).

Results: Incidence of ICH in patients with ITP was 1.540% in all ages, and 0.774% in those under age 18. After matching, 942 patients from each cohort were identified. Mean age was 58.3 years versus 61.2 years in cohort 1 and 2, respectively. Mortality rate was 34.076% vs. 20.17% (p <0.0001, OR 2.046 with 95% CI 1.661,2.520) at five years. Thirty-day survival was 83.46% vs. 95.17% (p<0.0001), and 365-day survival 68.59% vs. 85.33% (p<0.0001). PEG placement was seen in 21 (2.229%) patients in cohort 1, and less than 10 patients (<1.062%) in cohort 2 (p<0.0464). 2.442% vs. 0% underwent craniotomy (p<0.0001), palliative care was involved in 15.711% vs. 7.962% (p<0.0001), ICU care was seen in 27.389% vs. 11.783% (p<0.0001), with a mean ICU stay of 8.075 vs. 5.812 days (p=0.0537). 6.582% vs. 3.715% had PE (p=0.0049), 7.643% vs. 7.113% experienced MI (p=0.6595), 9.236% vs. 4.883% had DVTs (p=0.0002), 23.673% vs. 5.732% had seizures (p<0.0001), 9.023% vs. 6.582% suffered falls (p=0.0482), 7.537% vs. 3.503% suffered IS (p<0.0001), and 15.074% vs. 8.174% experienced other VTE (p<0.0001).

Conclusion: ICH occurs in approximately 1.54% of ITP patients, and is associated with a 34% mortality rate, increased PEG tube placement rates, palliative care involvement, ICU care, craniotomy, PE, IS, DVT, seizures, and falls.

## Introduction

Intracerebral hemorrhage (ICH) is a devastating complication of immune thrombocytopenic purpura (ITP). In 2009, Psaila et al. reported data from a national survey estimating ICH incidence was 0.19%-0.78% in children (age 17 or younger), with a mortality rate of approximately 25% [1]. We sought to investigate this claim in children, as well as the incidence in adults, using a global research network to retrospectively examine the incidence of ICH in patients with ITP while also looking at the mortality rate as a primary endpoint. Secondary endpoints include PEG tube placement, craniotomy, palliative care encounters, intensive care unit (ICU) management, seizure, falls, pulmonary embolism, myocardial infarction, deep venous thrombosis, ischemic stroke, splenectomy, and other venous embolism and thrombosis. 

## Materials and methods

This was a retrospective case-control study. We used a de-identified database network (TriNetX) to retrospectively query via International Classification of Disease (ICD-10) codes and procedural codes via Common Procedural Terminology (CPT) codes to evaluate all patients with a diagnosis of ITP and ICH (cohort 1) versus ITP alone (cohort 2). Data came from 63 health care organizations (HCOs) spanning 11 countries. Data includes demographics, diagnoses, medications, laboratory values, genomics, and procedures. The identity of the HCOs and patients are not disclosed to comply with ethical guidelines against data re-identification. Because of the database's federated nature, an IRB waiver has been granted. Our use of this database and its validity was informed by previous literature, and exact details of the network have been previously described [2-5]. Data is uploaded from each HCO data center. Some HCOs send data directly from the electronic health record, whereas others use data warehouses using known data models such as i2b2. Data is analyzed for cleanliness, consistency, correctness, and completeness according to established methodology [4]. Data spanned the years 2008-2021. The index date was set at the date of ITP and ICH (cohort 1) versus ITP alone (cohort 2). Medical information including age at index date, as well as sex, race, and comorbidities of hypertension, acute kidney injury, diabetes, ischemic heart disease, heart failure, atrial fibrillation, disorders of lipoprotein metabolism and other lipidemias, obesity, history of nicotine dependence, chronic respiratory disease, cirrhosis, alcohol abuse or dependence, peripheral vascular disease, and use of aspirin, warfarin, apixaban, and rivaroxaban were recorded up to the date of the index date. Analysis was performed using unmatched and propensity score-matched cohorts, with the greedy-nearest neighbor algorithm with a caliper of 0.1 pooled standard deviations. The primary endpoint was mortality, with secondary endpoints of PEG tube placement, craniotomy, palliative care encounters, intensive care unit (ICU) management, seizure, falls, pulmonary embolism, myocardial infarction, splenectomy, deep venous thrombosis, ischemic stroke, and other venous embolism and thrombosis. Hazard ratios were calculated using R's survival package v3.2-3 and validated comparing the output to that of SAS version 9.4 (SAS Institute Inc., Cary, NC, USA). Chi-square analysis was performed on categorical variables. Significance level was set at p<0.05.

## Results

A total of 948 patients with ITP and ICH were identified, and 57,887 with ITP and no ICH. After propensity score matching, 942 patients were identified in each cohort. After matching, average age at index was 58.3+-22.6 and 61.2+-21.8 (range 1-90) years for cohorts 1 and 2, respectively. 51.911% of cohort 1 were male, and 53.822% were in cohort 2. 68.577% vs. 68.79% of patients were White, 14.013% vs. 14.862% were Black or African American, and 1.38% vs. 2.017% were Asian. In addition, 5.732% vs. 6.582% of patients were taking aspirin, 1.805% vs. 1.911% were taking warfarin, and <10 patients in both cohorts were taking apixaban or rivaroxaban. Baseline demographics and characteristics are shown in Table 1.

**Table 1:** Baseline demographics and characteristics after propensity score matching Cohort 1: ITP and ICH. Cohort 2: ITP and no ICH ITP: immune thrombocytopenic purpura. ICH: intracerebral hemorrhage

		Before Matching			After Matching		
Code	Diagnosis	Cohort 1, n (%)	Cohort 2, n (%)	Std diff.	Cohort 1, n (%)	Cohort 2, n (%)	Std diff.
AI	Age at Index	58.35 (100.000)	46.52 (100.000)	-	58.26 (100.000)	61.19 (100.000)	-
2106-3	White	650 (68.565)	39613 (68.598)	0.000692	646 (68.577)	648 (68.790)	0.0046
M	Male	494 (52.110)	24759 (42.875)	0.185723	489 (51.911)	507 (53.822)	0.0383
F	Female	454 (47.890)	32959 (57.075)	0.1847	453 (48.089)	435 (46.178)	0.0383
2131-1	Unknown Race	149 (15.717)	10882 (18.844)	0.082777	148 (15.711)	133 (14.119)	0.0447
2054-5	Black or African American	133 (14.030)	5654 (9.791)	0.131137	132 (14.013)	140 (14.862)	0.0242
2028-9	Asian	13 (1.371)	1301 (2.253)	0.06613	13 (1.380)	19 (2.017)	0.0493
I10-I16	Hypertensive diseases	452 (47.679)	12569 (21.766)	0.565656	446 (47.346)	476 (50.531)	0.0637
N17-N19	Acute kidney failure and chronic kidney disease	250 (26.371)	5508 (9.538)	0.449522	247 (26.221)	260 (27.601)	0.0311
E78	Disorders of lipoprotein metabolism and other lipidemias	216 (22.785)	7235 (12.529)	0.271439	212 (22.505)	224 (23.779)	0.0302
E08-E13	Diabetes mellitus	189 (19.937)	6013 (10.413)	0.26783	185 (19.639)	216 (22.930)	0.0805
I20-I25	Ischemic heart diseases	186 (19.62)	4499 (7.791)	0.349174	182 (19.321)	206 (21.868)	0.0630
I50	Heart failure	148 (15.612)	2820 (4.883)	0.359422	145 (15.393)	153 (16.242)	0.0233
I48	Atrial fibrillation and flutter	145 (15.295)	3159 (5.47)	0.326349	143 (15.180)	157 (16.667)	0.0406
R53	Malaise and fatigue	119 (12.553)	2012 (3.484)	0.338675	117 (12.420)	128 (13.588)	0.0347
Z87.891	Personal history of nicotine dependence	115 (12.131)	2702 (4.679)	0.271024	111 (11.783)	128 (13.588)	0.0542
J40-J47	Chronic lower respiratory diseases	111 (11.709)	4520 (7.827)	0.131026	111 (11.783)	114 (12.102)	0.0098
R40	Somnolence, stupor and coma	115 (12.131)	342 (0.592)	0.486552	109 (11.571)	80 (8.493)	0.1026
E65-E68	Overweight, obesity and other hyperalimentation	93 (9.810)	3190 (5.524)	0.161612	91 (9.660)	87 (9.236)	0.0145
R13	Aphagia and dysphagia	87 (9.177)	674 (1.167)	0.367748	83 (8.811)	83 (8.811)	0.0000
F17	Nicotine dependence	58 (6.118)	2505 (4.338)	0.080043	58 (6.157)	60 (6.369)	0.0088
K74	Fibrosis and cirrhosis of liver	35 (3.692)	1299 (2.249)	0.085041	35 (3.715)	29 (3.079)	0.0352
I73	Other peripheral vascular diseases	35 (3.692)	904 (1.565)	0.133213	33 (3.503)	39 (4.140)	0.0332
R63	Symptoms and signs concerning food and fluid intake	22 (2.321)	648 (1.122)	0.092245	22 (2.335)	36 (3.822)	0.0861
F10.1	Alcohol abuse	20 (2.110)	404 (0.700)	0.120038	20 (2.123)	21 (2.229)	0.0073
F10.2	Alcohol dependence	20 (2.110)	433 (0.750)	0.114739	20 (2.123)	18 (1.911)	0.0151
1191	Aspirin	54 (5.696)	2082 (3.605)	0.09941	54 (5.732)	62 (6.582)	0.0353
11289	Warfarin	17 (1.793)	553 (0.958)	0.071792	17 (1.805)	18 (1.911)	0.0079
1364430	Apixaban	<10 (<1.055)	226 (0.391)	0.078369	<10 (<1.062)	<10 (<1.062)	0.0000
1114195	Rivaroxaban	<10 (<1.055)	151 (0.261)	0.098234	<10 (<1.062)	<10 (<1.062)	0.0000

Incidence of ICH in patients with ITP was 1.540% in all ages, and 0.774% in those under age 18. Mortality rate was 34.076% in cohort 1 vs. 20.17% in cohort 2 (p <0.0001, OR 2.046 with 95% CI 1.661,2.520), respectively. Thirty-day survival was 83.46% vs. 95.17% (p<0.0001), 90-day survival 76.98% vs. 90.44% (p<0.0001), and 365-day survival 68.59% vs. 85.33% (p<0.0001). PEG tube placement was seen in 21 (2.229%) patients in cohort 1, and less than 10 patients (<1.062%) in cohort 2 (p<0.0464). 2.442% in cohort one underwent craniotomy, vs. 0% in cohort 2 (p<0.0001), palliative care was involved in 15.711% vs. 7.962% of patients (p<0.0001), ICU care was seen in 27.389% vs. 11.783% (p<0.0001), with a mean number of days of 8.075 vs. 5.812 (p=0.0537). Sixty-two patients (6.582%) experienced pulmonary embolism in cohort 1, vs. 35 (3.715%) in cohort 2 (p=0.0049), 7.643% vs. 7.113% experienced a myocardial infarction (p=0.6595), 9.236% vs. 4.883% had DVTs (p=0.0002), 23.673% vs. 5.732% had seizures (p<0.0001), 9.023% vs. 6.582% suffered falls (p=0.0482), 7.537% vs. 3.503% suffered ischemic stroke (p<0.0001), and 15.074% vs. 8.174% experienced other venous embolism and thrombosis (p<0.0001).

**Figure 1:**
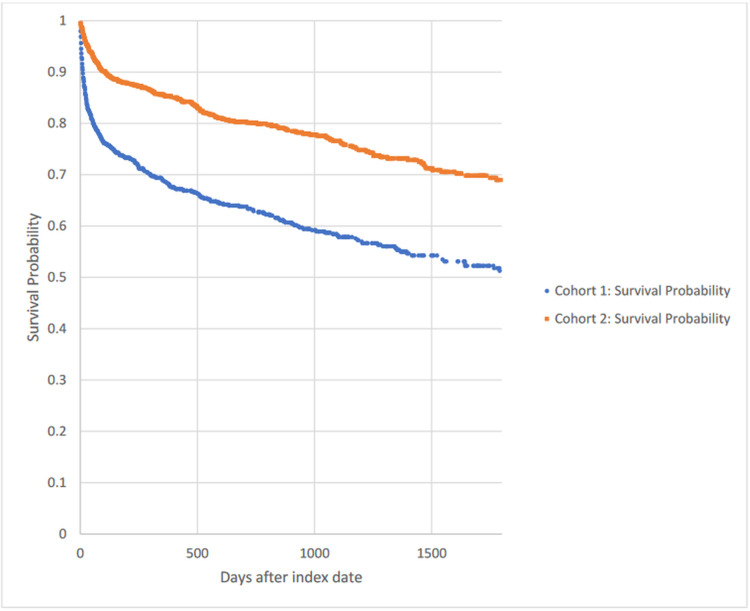
Kaplan-Meier survival analysis for outcome: deceased Cohort 1: ITP and ICH. Cohort 2: ITP and no ICH ITP: immune thrombocytopenic purpura. ICH: intracerebral hemorrhage

Figure 1 shows a Kaplan-Meier survival curve for outcome decreased comparing cohorts 1 and 2. The hazard ratio was 2.077, with 95% CI 1.735,2.486, p<0.0003.

Table 2 shows outcomes after propensity score matching.

**Table 2:** Outcomes after propensity score matching. Cohort 1: ITP and ICH. Cohort 2: ITP and no ICH ITP: immune thrombocytopenic purpura. ICH: intracerebral hemorrhage. PEG: percutaneous endoscopic gastrostomy

Outcome	Cohort 1, n (%)	Cohort 2, n(%)	Odds ratio (95% CI)	P-value
Mortality	321 (34.076)	190 (20.17)	2.046 (1.661,2.520)	<0.0001
PEG	21 (2.229)	<10 (<1.062)	2.125 (0.995,4.537)	<0.0464
Craniotomy	23 (2.442)	0 (0.000)	-	<0.0001
Palliative care	148 (15.711)	75 (7.962)	2.155 (1.606,2.891)	<0.0001
Intensive care unit care	258 (27.389)	111 (11.783)	2.824 (2.212,3.606)	<0.0001
Pulmonary embolism	62 (6.582)	35 (3.715)	1.826 (1.194,2.792)	0.0049
Myocardial infarction	72 (7.643)	67 (7.113)	1.081 (0.765,1.527)	0.6595
Deep venous thrombosis	87 (9.236)	46 (4.883)	1.982 (1.370,2.868)	0.0002
Splenectomy	14 (1.486)	<10 (<1.062)	1.406 (0.621,3.182)	<0.4112
Seizure	223 (23.673)	54 (5.732)	5.1 (3.729,6.976)	<0.0001
Fall	85 (9.023)	62 (6.582)	1.408 (1.001,1.979)	0.0482
Ischemic stroke	71 (7.537)	33 (3.503)	2.245 (1.471,3.429	0.0001
Other venous embolism and thrombosis	142 (15.074)	77 (8.174)	1.994 (1.487,2.674)	<0.0001

## Discussion

In this retrospective case-control study, we assessed the incidence, morbidity, and mortality of ICH following the diagnosis of ITP in all ages utilizing data, including ICD-10 codes from a de-identified database spanning over 11 countries and 63 healthcare organizations. After propensity-score matching, we were able to gather data on two large cohorts with more than 900 patients. We controlled for variables, including age, sex, race, anticoagulation use, and common comorbidities to reduce potential confounding.

Our study reports an incidence of ICH in all ITP patients of 1.540% (0.770% in patients under 18). The incidence of this grave complication continues to remain a dubious topic in the literature. Our findings, specifically for patients under 18 (0.770%), fall within the range that Psaila et al. reported in their retrospective study (0.19-0.78) [1]­­­­­­­ and reflect the rates seen in a recent prospective study by Schifferli et al. (0.6% of ICH in patients under 18 with ITP, 1.7% ICH in adult patients with ITP) [6]. Further stratification of ICH incidence in ITP patients within both pediatric and adult populations continues to be reported in wide ranges. This is likely due to the varying parameters in retrospective and prospective studies, respective cohort sizes, and methodologies that have been utilized in the literature [7].

Compared to other database studies, our study reports a higher incidence of ICH in ITP patients. Bhatt et al. observed trends in the National Inpatient Sample (NIS) database using ICD codes from 2007-2016 and reported an overall incidence of 1.1% in all patients and 0.7% in pediatric patients [8]. Danese et al. noted the incidence of 1.5% in adults from 2003-2006 [9]. An and Wang published outcomes of adult hospitalizations with ITP in the US from 2006 to 2012 and found the incidence of ICH at 1.28% [10]. Despite the wide variation of incidence among the literature with varying methodology, our analysis supports the overall summation and tone of the literature: ICH continues to remain rare but a grave complication of ITP in both pediatric and adult populations associated with a higher level of care, significant adverse events, and mortality.

Mortality for patients with ITP alone was found to be 20%, comparable to other mortality rates reported in patients with ITP [8]. Those who experienced the complication of ICH had an overall mortality rate of 34% and significantly higher mortality at 30-, 90-, and 365-day time points (p<0.0001) compared to ITP patients without ICH. Thus, ITP patients with ICH are twice as likely to experience mortality than patients with ITP alone. ICD-10 codes for ICU level of care and palliative care consultation were pursued more for ICH ITP patients as well (p<0.0001). Bhatt et al. in 2020 reported an overall mortality of 26.7% in ITP patients with ICH within their database study. They additionally report a longer length of stay (LOS) for ITP patients with ICH, which correlates to our findings and increased hospitalization cost [8].

Interventions such as craniotomy and PEG placement were pursued in 2.4% and 2.2%, respectively, in ITP patients with ICH compared to no invasive cranial intervention and 1.1% PEG placement in patients with ITP alone. Regarding neurological sequelae, the rate of seizures and falls were significantly higher in ITP patients who experienced ICH, reflecting the critical nature of this diagnosis and potential sequelae from it.

ICH in ITP patients was associated with a significantly elevated risk of thromboembolic events: ischemic stroke (p=0.0001), pulmonary embolism (p=0.0049), DVT (p=0.0002), and other thromboembolic events (p=<0.0001). The literature documents that despite low platelet count in ITP patients, thrombocytopenia does not prevent thromboembolic disease. The pathophysiology of arterial and venous thromboembolic events in ITP patients remains elusive [11,12]. ITP is a condition involving low platelet counts, but hypofunctioning and hyperfunctioning platelets; platelet microparticles (PMPs) continue to remain the longstanding hypothesis encompassing a potential mechanism within this paradoxical presentation; these PMP levels were found to be significantly elevated in ITP patients who experienced neurologic complications and had confirmed CT imaging revealing small cerebral infarcts [13]. There are currently no clinical guidelines regarding the management of thrombotic events in ITP patients [14], and current literature is limited to case reports, case series, and small cohort studies involving the complex judgment of administering anticoagulation in a patient with impaired coagulation and increased risk of bleeding [15-19]. Thrombopoietic therapies have been pursued and utilized for management of ITP [20]; our findings specifically for ITP patients with ICH may raise caution for the administration of these agents given the significant risk for prothrombotic events in this population. Further research on the pathophysiology and management of this clinical challenge is needed.

Our analysis was not without limitations. The major limitation of this study was that it was retrospective in nature. Furthermore, due to the nature of the database, we were unable to collect patient-level data on specific outcomes. We were unable to report on radiology information. We do not have information on the type of diagnostic test used for confirmation of disease. The data collected was for billing purposes, not for clinical use, and thus much clinical information is missing. In addition, some misidentification is inevitable in database studies.

## Conclusions

ICH occurs in approximately 1.54% of ITP patients. ITP with ICH is associated with an approximately 34% mortality rate, increased PEG tube placement rates, palliative care involvement, ICU care, craniotomy, pulmonary embolism, ischemic stroke, DVT, seizures, and falls. 
